# Variability in the Calculation of Time in Therapeutic Range for the Quality Control Measurement of Warfarin

**DOI:** 10.19102/icrm.2018.091203

**Published:** 2018-12-15

**Authors:** Safia Siddiqui, Christina E. DeRemer, Jennifer L. Waller, Jaspal S. Gujral

**Affiliations:** ^1^Department of Medicine, Augusta University, Augusta, GA, USA; ^2^College of Pharmacy, University of Florida, Gainesville, FL, USA

**Keywords:** Anticoagulant agents, INR, quality control, quality improvement, warfarin

## Abstract

Time in therapeutic range (TTR), a well-recognized performance metric of oral anticoagulation, measures the time when patients’ international normalized ratios (INRs) are within the desired range. The TTR value can vary significantly depending on the type of method used and can be a skewed indicator of the overall quality of anticoagulation. As such, the present study was designed to compare three methods for TTR calculation (cross-sectional, traditional, and Rosendaal) to quantify their differences, biases, and trends. As part of this investigation, a 21-week retrospective analysis of patients on warfarin was conducted to compare TTR values obtained by these three methods. Paired t-tests, correlation studies between size and bias, and Bland–Altman plots were performed using SAS 9.4 (SAS Institute, Cary, NC, USA). It was revealed that the TTR values for the cross-sectional, Rosendaal, and traditional methods were 65.97, 58.12, and 51.55, respectively. The addition of tolerances to INR ranges of ± 0.2 and ± 0.5 increased TTR values to 81.79 and 91.53, respectively, for the cross-sectional method, and 66.86 and 82.69, respectively, for the traditional method. The use of the traditional method resulted in significantly higher TTR values than did use of the Rosendaal method, with high variability between the methods in both positive and negative directions. There was a demonstrated lack of independence between the methods, and zero bias could not be assumed. In conclusion, the different methods considered in the present study do not accurately measure whether a patient is in or out of the therapeutic range, and the addition of tolerances can further distort the perception of anticoagulation achieved. We recommend a standardized TTR calculation method as well as a uniform tolerance for use in clinical trials and quality control efforts.

## Introduction

Warfarin is a highly effective anticoagulant backed by an accumulation of data from more than 60 years of clinical experience and practice familiarity. Currently, it is still considered the most commonly prescribed oral anticoagulant despite the release of direct oral anticoagulants (DOACs) due to several factors such as gaps in clinical indications studied, concerns of limited reversibility, and cost.^1^ DOACs are considered pharmacokinetically reliable agents, and, therefore, their monitoring is not an expectation; however, many providers or institutions also lack the resources to monitor and interpret the results of using these drugs. Thus, warfarin has maintained a strong presence in clinical practice.

Time in therapeutic range (TTR) is interpreted to be a reflection of the overall quality of anticoagulation achieved on warfarin in clinical practice as well as in pharmaceutical trials. Separately, the international normalized ratio (INR) is a standard test used by providers for patients who are on anticoagulation therapy. Per guidelines, the INR allows for a provider to evaluate the immediate control of warfarin dosage and adjust it accordingly. TTR, by taking into account multiple INR values from contiguous visits, is used to reflect anticoagulation control over time. TTR can be used to measure INR control over time for an individual patient or for a patient population.

There are three commonly accepted methods for calculating TTR; these are the Rosendaal 
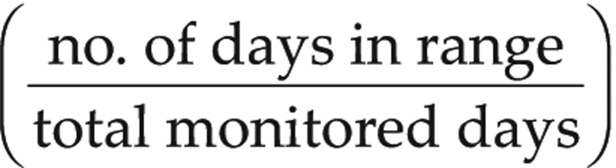
, traditional 
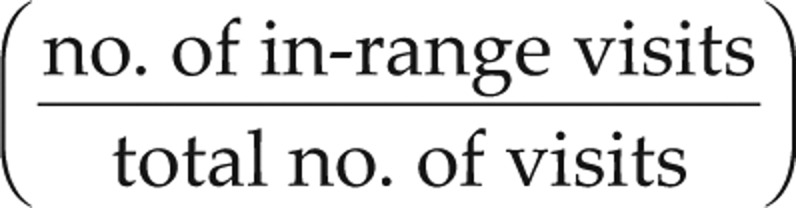
, and cross-sectional 
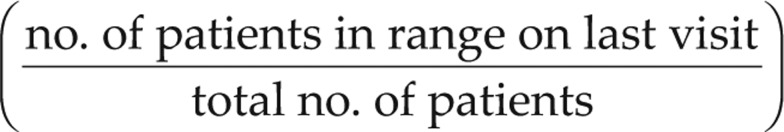
 methods. The Rosendaal method assumes a linear progression of change in INR between a patient’s visits; in other words, it assumes that the INR changes the same amount each day.^2^ Meanwhile, the traditional and cross-sectional methods do not treat INR as a dynamic value that changes over time. Instead, these methods consider each individual INR value to be static and binary, either in or out of range. The traditional method calculates TTR as the proportion of in-range INR values to the total number of INR values, whereas the cross-sectional method takes into account only the INR from the last visit before an arbitrarily chosen date.^3^ Each method, due to its design and inherent assumptions, can yield significantly different TTR values. Moreover, tolerance levels of ± 0.2 or ± 0.5 are often applied (to the endpoints of an individual’s goal INR range) to widen the therapeutic INR window.^4^ The resultant TTR value depends heavily on both the specific method and the tolerance level used and can thus provide a misleading representation of anticoagulation achieved, be it in clinical practice or in a controlled trial.

The present project was initiated as part of an effort to compare the three methods and quantify their differences, biases, and trends. By applying each method with different tolerance levels to patient data and analyzing the resultant TTR values, we hope to gain a better understanding of the inherent shortcomings of each method. The importance of having this understanding lies in determining its effect on the interpretation of controlled trials that reference TTR and in comprehending its use to compare the effectiveness of anticoagulant agents.

## Materials and methods

### Study patients and methodology

This study was a 21-week retrospective analysis that compared TTR results obtained using the three aforementioned TTR calculation methods for patients monitored on warfarin at a pharmacist-managed anticoagulation clinic in an academic medical center. All patients who had at least two INR values, each from a separate visit and obtained within the defined study period, were included. Patients were required to have been enrolled in the clinic for at least 30 days before the study’s start date. On average, patients visited the clinic every two weeks. The INR was measured using a finger-prick point-of-care device, the Hemochron^®^ Signature Elite (Accriva Diagnostics, San Diego, CA, USA), which outputs an “error” response for an INR value of more than 10. If the point-of-care INR was out of therapeutic range for the patient or if it outputted an “error” value, the measurement was repeated using a venous blood sample. If the repeated measure was still out of the therapeutic range following the use of the venous blood sample, then the patient’s warfarin dosage was adjusted accordingly as per standardized protocols. Patients were excluded if they had only one visit within the study period. Patient data were extracted from PowerChart^®^ (Cerner, North Kansas City, MO, USA), deidentified, and recorded in Microsoft Excel (Microsoft Corp., Redmond, WA, USA).

### Statistical methods

All statistical analyses were performed using SAS 9.4 (SAS Institute, Cary, NC, USA), and statistical significance was assessed using an alpha level of 0.05. Descriptive statistics for each method, frequencies, and proportions for the cross-sectional method as well as means and standard deviations for the traditional and Rosenthal methods were calculated. A histogram of the percentage at each TTR calculation for both the traditional and Rosendaal methods was plotted for descriptive purposes. To examine whether the individuals were considered to be in range greater or less than 50% of the time when using the cross-sectional method (using the method as is and using the method with a tolerance of ± 0.2 or ± 0.5 to the endpoints of the individual’s goal range), a one-sample test of a binomial proportion was used. Additionally, to compare the means of the traditional and Rosendaal methods between those classified as in or out of range by the cross-sectional method, two-sample t-tests were used. To compare the traditional (using the method as is and using the method with a correction of ± 0.2 or ± 0.5 to the endpoints of the individual’s goal range) and the Rosendaal methods, several different analyses and plots were performed. First, scatterplot of the traditional method versus the Rosendaal method were created with the identity line (X = Y) as a reference line. Second, paired t-tests between the two methods were used to examine whether there was zero bias between the methods. Third, the difference between the two methods [calculated as traditional minus Rosendaal (T − R)] and the size of the TTR of the two methods [(T + R / 2)] were calculated. The correlation between the difference and the size of the TTR was examined to determine whether there was independence between the bias (the difference) and the size (the mean) of the methods. Fourth, a Bland–Altman plot, which plots the difference between the two methods relative to the size of the two methods for each individual, was used to assess the magnitude of disagreement (in both error and bias), identify spot outliers, and see whether there was any trend in the bias relative to the size of the TTR determined by the methods used.

## Results

**Figure 1:**
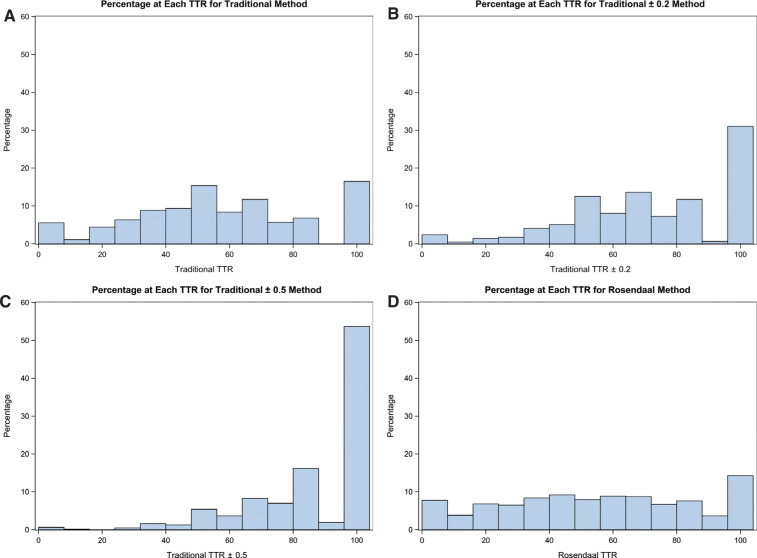
Percentage at each TTR point for the **(A)** traditional, **(B)** traditional ± 0.2, **(C)** traditional ± 0.5, and **(D)** Rosendaal methods. TTR: time in therapeutic range.

A total of 612 patients met the inclusion criteria and were included in the data analysis. All cross-sectional TTR methods gave a percentage in the range that was significantly greater than 50%, as noted in **Table 1**. **Table 2** presents the descriptive statistics for the traditional and Rosendaal methods as well as for the difference (T − R) and the average size (T + R / 2) of the two methods. Using the traditional TTR method without tolerance versus the Rosendaal method gave similar mean TTR values of 56.6 and 55.1, respectively. Expanding the goal range by ± 0.2 or by ± 0.5 for the traditional method resulted in increased TTR values of 71.4 and 85.7, respectively. **Figure 1** shows the relative frequency (percentage) of patients at each TTR calculation for the traditional and Rosendaal methods.

**Figure 2:**
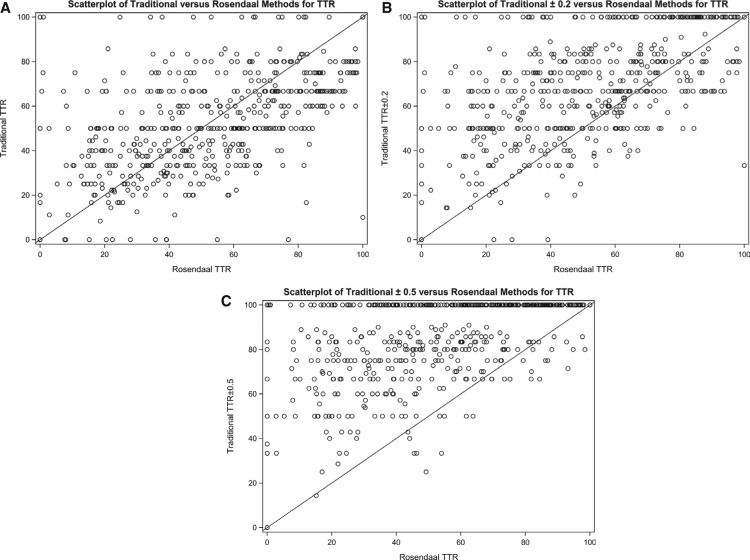
Scatterplots of the Rosendaal method versus the **(A)** traditional, **(B)** traditional ± 0.2, and **(C)** traditional ± 0.5 methods. TTR: time in therapeutic range.

**Figure 2** shows the scatterplots of the Rosendaal versus the traditional methods. There is little indication from these scatterplots that the methods are comparable given the scatter in both the X and Y directions in the plots. Data should fall along the X = Y identity line for methods that may be considered comparable.

Paired t-tests of the traditional and Rosendaal methods are presented in **Table 3** and were used to examine whether zero bias between the two methods can be assumed. When comparing the traditional method without an adjustment to the INR goal to the Rosendaal method, there was zero bias between the two methods. However, adding ± 0.2 or ± 0.5 to the goal for the traditional method resulted in a lack of zero bias with the traditional method, giving rise to significantly higher TTR values as compared with in the case of the Rosendaal method.

**Table 4** examines the correlation between the bias (the difference between T − R) and the size (T + R / 2) of the traditional and Rosendaal methods. Although all correlations were statistically significant, indicating a lack of independence, using the traditional method as is had a lower correlation than when adding ± 0.2 or ± 0.5 to the goal.

**Figure 3:**
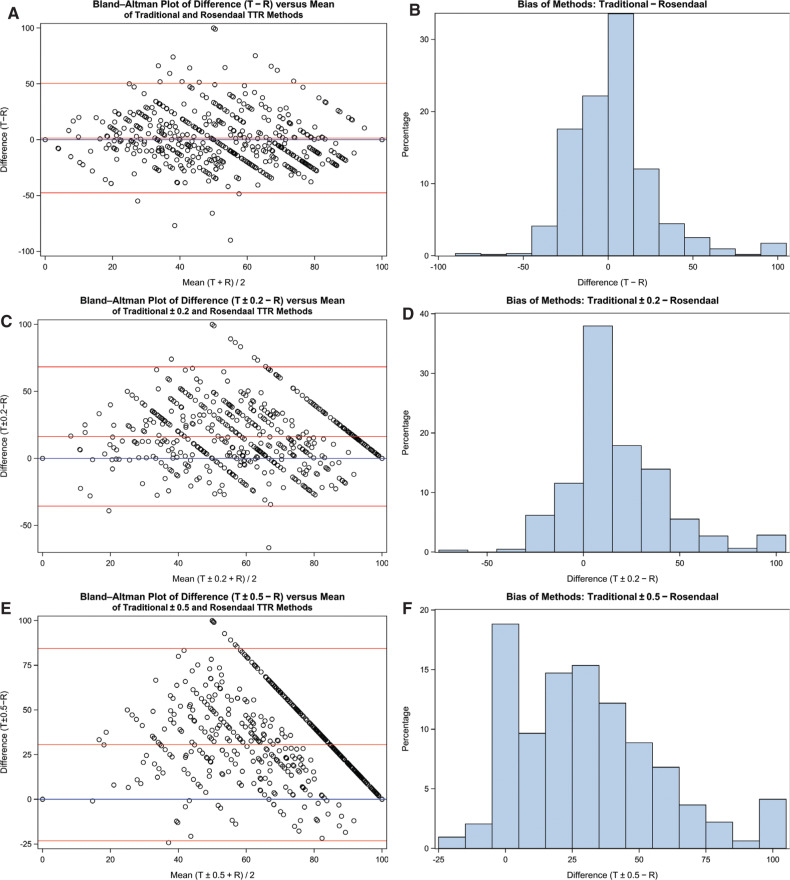
Bland–Altman plots for difference (T − R) versus size (T + R / 2) with frequency of bias plots. The blue line represents zero difference; the red lines represent mean difference ± two × standard deviation of the difference. **A and B:** Traditional and Rosendaal; **C and D:** traditional ± 0.2 and Rosendaal; **E and F:** traditional ± 0.5 and Rosendaal. TTR: time in therapeutic range; T: traditional; R: Rosendaal.

Upon examination of the Bland–Altman and bias plots in **Figure 3**, there appeared to be high variability between the traditional (used as is) and the Rosendaal methods when the TTR value was between 35% and 65% (note the diamond-shaped pattern). The traditional method tends to have higher TTR values versus the Rosendaal method, as can be seen in the bias plot, where there are more positive differences than negative differences. This pattern of bias shifts to the right and upward when adding ± 0.2 or ± 0.5 to the goal for the traditional method, with the traditional method having higher TTR values when compared with the Rosendaal method.

## Discussion

TTR is a widely used quality control measure that is directly correlated with therapeutic effectiveness and the minimization of adverse outcomes.^5^ The TTR values noted in our patient population are comparable to values presented previously in the literature, ranging from 25% to 65%, with an average of 50% to 55%.^6^ However, the actual result, and therefore the interpretation of anticoagulation achieved, is directly related to the type of method used to calculate the TTR. With the addition of tolerances and modifications to time of enrollment, values can be even further skewed. Per the one sample t-test of binomial proportion, the cross-sectional method resulted in a TTR value significantly higher than 60%, even without any added tolerance.

The paired t-tests comparing the traditional and Rosendaal methods showed zero bias when no tolerance was added to the traditional method. Once tolerance was added, the traditional method showed significantly higher values than did the Rosendaal method, which could be misinterpreted as indicating better management on warfarin. The statistically significant correlations between size and bias of the traditional and Rosendaal methods seen specifically when adding tolerance showed a lack of independence, indicating that, as the size of the measure increased, the bias between the measures decreased. The lower correlation result obtained using only the traditional method (without tolerance) indicates that adding tolerance could contribute to a lack of independence between the methods. Schmitt et al.^7^ found similar t-test results among the three methods, but their analysis did not include bias, difference, or variability or consider the effect of adding tolerances to each.

Although the TTR values obtained by the Rosendaal method appeared more uniformly distributed across our sample population, this method tends to underestimate TTR values in comparison with the traditional method. As expected, adding tolerance to the traditional method skews TTR results toward higher values **(Figure 1)**. The scatterplots of the traditional method versus the Rosendaal method showed high variability, which increased even more as tolerance was added to the traditional goal ranges. If the methods were comparable, the data would be expected to fall along the X = Y line. However, the distribution of data points clearly indicate huge variability that worsens with added tolerance.

The Bland–Altman plots demonstrated high variability between the traditional (without tolerance) and Rosendaal methods when the TTR value was between 35% and 65%. As tolerances are added and increased, the reliability of TTR as an accurate measure of anticoagulation is diminished. This visually illustrates the limitations for applications to clinical practice. The traditional method tends to have higher TTR values than does the Rosendaal one, with an even larger discrepancy seen upon the addition of tolerances. The large amount of scatter in the differences relative to the mean indicates that the methods are not providing similar measurements. Thus, comparison across clinical trials does not measure similarly analyzed data and therefore should not be compared.

The bias bar graphs clearly indicate that, once tolerances are added, more patients are skewed to a higher TTR value. The resulting interpretation thus is better control and management of warfarin that is not reflected in actual clinical practice. Again, the traditional method tends to have higher TTR values as compared with the Rosendaal method, as demonstrated by the positive differences.

It has been suggested that the incorporation of more variables in the calculation of TTR, such as in the TTR-F formula proposed by Reiffel,^8^ would improve its accuracy as a quality control measure. However, the level of detail needed (such as mean INR, number of INR measurements, and percentage of INR out of range) raises concerns regarding the expected high level of complexity and the time required, which may serve as a barrier to its usage.

### Study limitations

Although there was a demonstrated lack of independence between the size and bias of the traditional and Rosendaal methods, this could be attributed to the sample size. The time of collection within the selected patient population was variable. Although some patients were closely followed at two-week intervals, others were monitored via monthly or bimonthly visits. This lack of standardization during the time of collection could be a confounding factor to the resultant TTR values. Data on interruptions in anticoagulation, whether planned or unplanned, were not collected, and all INR measures that met the inclusion criteria were made part of the study. Another limitation is the smaller study time frame of 21 weeks, which could have affected the resultant TTR values.

## Conclusion

Our analyses demonstrate that all three methods have wide variability in comparison with one another. No method appears to be better than another; however, the Rosendaal method is the only method specifically designed for INR control over time and is used more widely in the literature. Although we support the use of TTR to measure the quality of anticoagulation management, the specific method used changes the resultant TTR and can distort the provider’s perception of anticoagulation achieved. Thus, we recommend disclosure of the method used to calculate TTR, of tolerances if they are being added to goal ranges, and of any modifications made to the time of collection. To avoid TTR results that falsely imply control that is absent from practice, we recommend the standardization of a TTR method and the tolerances used in clinical trials and practice management efforts. This would allow for fair comparisons between trials as well as the evaluation of the quality of anticoagulation in practice.

## Figures and Tables

**Table 1:** Descriptive Statistics for Percentage in Range for the Cross-sectional TTR Method and Binomial Test for Difference from 50%

Method	Percentage in Range	95% Confidence Interval	p-value
Cross-sectional	66.0%	62.3–69.7	0.0023
Cross-sectional ± 0.2	81.8%	78.8–84.8	< 0.0001
Cross-sectional ± 0.5	91.5%	89.1–93.6	< 0.0001

**Table 2:** Descriptive Statistics for the Traditional and Rosendaal TTR Methods, Differences (T − R), and Size (T + R / 2)

Method	Mean	Standard Deviation	95% Confidence Interval
Traditional	56.5	27.8	54.4–58.7
Traditional ± 0.2	71.4	25.5	69.4–73.4
Traditional ± 0.5	85.7	19.4	84.2–87.2
Rosendaal	55.1	30.2	52.7–57.5
Differences (T − R)			
Traditional − Rosendaal	1.5	24.5	−0.5 to 3.4
Traditional ± 0.2 − Rosendaal	16.3	26.0	14.3–18.4
Traditional ± 0.5 − Rosendaal	30.6	26.9	28.5–32.7
Size (T+R / 2)			
Traditional + Rosendaal	55.8	26.3	53.8–57.9
Traditional ± 0.2 + Rosendaal	63.3	24.7	61.3–65.2
Traditional ± 0.5 + Rosendaal	70.4	21.5	68.7–72.1

**Table 3:** Paired t-tests of Traditional versus Rosendaal Methods to Assess Zero Bias between the Two TTR Methods

Method	Mean Difference	Standard Deviation of Difference	t-statistic	p-value
Traditional – Rosendaal	1.45	24.5	1.49	0.1366
Traditional ± 0.2 – Rosendaal	16.3	26.0	15.79	< 0.0001
Traditional ± 0.5 − Rosendaal	30.6	26.9	28.62	< 0.0001

**Table 4:** Correlation of Difference with Size of the Methods to Assess Independence

Method	r-value	p-value
Traditional and Rosendaal	−0.1096	0.0058
Traditional ± 0.2 and Rosendaal	−0.2056	< 0.0001
Traditional ± 0.5 and Rosendaal	−0.4655	< 0.0001
